# Indents

**Published:** 1907-02-15

**Authors:** 


### INDENTS.

**Brief Articles of Technical or Scientific Value will receive notice and comment. Contributions invited.**

**Testing an Electric Furnace.** Every user of an electric furnace should provide for testing his muffle in case of failure to operate. This is easily done by taking a few feet of the ordinary electric light wire connected with a plug at one end and a small copper or brass point on each of the other ends. One of these wires should be cut and a lamp put in. The lamp will light up when points are brought into contact with the two terminal wires in the muffle if the wire is not burned out or broken. Some such arrangement will also prove to be a very great help in locating a break in case of repair. Where the wire has burned out there will usually appear a spot, but a break will not show and a tester is indispensable. In the majority of cases the burn will occur within three or four inches of the negative end of the wire, due to the excessive heating of the wire at that point.—F. E. Roach, *Dental Review.*

**How the Ancients Ate.** A Constantinople paper prints extracts from an ancient Turkish volume recently discovered which deals with the table manners of bygone centuries. Many of the directions still serve for modern western nations. Here are some of the receipts:

"To sit down to dinner alone is to be avoided, the prophet always dined in company." "Food should be conveyed to the mouth with the right hand." "Before and after meal swallow a little salt." "Eat not thy bread with the knife, but break it with the hands." "Thou shalt not place a dish on a piece of bread nor wipe thy fingers on bread." "When a piece of bread falls to the ground pick it up carefully." "At table thou shalt comport thyself decently with dignity, not lean lazily on the cloth, for so sayeth the prophet." "When the meal is over collect the crumbs, wash thy hands and mouth, clean thy teeth and render thanks to Allah"—*Free Press*,

**Sensitive Dentin.** Dr. E. A. Bogen, speaking before the American Dental Society of Europe, said his own practice was a very limited one and he did not suppose he encountered a hundredth part of the difficulties encountered by his professional brethren engaged in a different field, but for eighteen or twenty years he had used a method which had never failed him. Chloride of zinc was advocated years ago and he tried it, but abandoned it on account of the pain it caused. Then it struck him that if he used carbolic acid as strong as it could be used, and by that means sufficiently solidified the albumenoids contained in the dental tubuli, he could use chloride of zinc in crystals and allow it to deliquesce. He did so, and he had been doing it for eighteen or twenty years, and not withstanding the statement made by some of the learned professors he had not succeeded in killing one single pulp.—Report in December 1906 *Review*.

**Copper for Matrices.** Obtain a matrix in ordinary manner using thin copper plate. For small cavities fuse matrix neariy full of 18-k. gold solder by holding in flame of the alcohol lamp. Replace in cavity and burnish well to cavity margins: remove and add solder until flush with margins. For large inlays, it is necessary to invest.

Place a *fold of small copper wire in bottom of matrix*, and run up with gold solder, using plenty of flux; grind nearly complete before setting and, last of all, drop the inlay into nitric acid to remove the copper. The copper wire having been removed by the acid leaves a good retaining groove.

Do not grind after removing from the acid until after inlay is set, as there would be danger of injuring the sharp marginal edges. These edges should be carefully burnished to the tooth while cement is soft. There are advantages in this method more patent than simply the price of the material.—V. B. Newell, *Dental Review.*

**Replacing Facings.** Remove the remains of the old facings and pins. Select a suitable facing, cut pinholes in the backing, and grind the facing to fit the backing accurately. Remove the facing, take a thin strip of platinum, and bend it around the pins to form a tube by means of a pair of flat pliers. Cut off the pins close to the tooth, adjust the platinum tubes, and solder each tube either by means of the blowpipe after investing the piece, or in a porcelain furnace, using the smallest possible quantity of solder. Slightly enlarge and countersink the holes in the backing; readjust the facing; hold it in position and burnish out the tube to fill the countersunk holes with a round burnisher. Then fill the opening with either gold or amalgam. The chief advantage of this method is in the ease with which facings are replaced in almost inaccessible places in the mouth and in that it permits of the use of saddle-back teeth so suitable for obtaining artistic effects.—W. A. Burns, January 1907, *Cosmos.*

**Ethyl Chlorid Fatalities.** Dr. Luke, in the *Lancet* for May 5th, 1906, says he had endeavored to roughly estimate the number of times Ethyl Chloride had been administered so that the percentage of fatalities might be figured to compare with chloroform and other anaesthetics. He then estimated that it had been administered 450,000 times in England. Since then he finds that one firm in London had sold enough for one and a half million administrations of five cubic centimeters each, and four other firms had sold enough to provide for three million administrations. And since only twenty deaths have been reported in England the mortality has been one in one hundred and fifty thousand. A much better record than chloroform. He adds, with these figures before us I am at a loss to understand how certain anaesthetists of repute steadfastly refuse to employ Ethyl-Chloride, alone or in combination. I am confident that it has saved many lives, or at least prevented fatalities which would have occurred had chloroform been used.—*Record*, January, 1907.

**Decent Cleanliness.** We've had lectures on sterilization and prophylaxis galore, but there's one important point, I think in connection with these subjects which has not been given its share of attention. That is our operating room. If you would have a perfect dental surgeons' operating room you would want a place with plenty of light good ventilation and furnished with only the articles necessary for dental use. I've seen a number of these so-called swell operating rooms, which looked to me more like a picture gallery and play house combined than anything else.

On the floor should be linoleum, which makes the best of of covering, and which also can be scrubbed and cleaned without raising dust particles and bacterial deposits which are sure to abound in carpets and rugs. The walls need not be decorated with pictures, as these catch dust deposits.

Use napkins on the head-rest of your chairs and don't get frightened if your laundry bill is high. The scrub woman's bill may also be high, but remember, Dr. Operator, everything connected with dentistry should be so very clean that you couldn't better it if you tried—*Odontoblast.*

**Gold Inlay.** When the Perfection compound in its little metal tray has been pushed into the cavity in the tooth, it is hardened with a jet of cold water from the syringe, and left in place a few moments until a mix of plaster is made. Now, with a partial-impression tray adapted to the needs of the case, a plaster impression is taken right over the little compound impression, and the imprint of the cavity proper will come out with the plaster if the cavity has been properly prepared. A wax bite is next taken, and the patient is allowed then to go. Now pack soft amalgam or flow your improved Spence metal, using only enough to give a die suitable to swage upon later. Varnish your plaster impression and run the cast with plaster in the usual manner. When hard, separate, and you have a perfect cast showing the adjoining teeth, and in this cast—as a part of it, in fact—one has the amalgam die which is to be the working model in the construction of the gold inlay. Now, with the aid of the wax bite which was taken at the first sitting, get up your articulation. With this model and articulation the operator is enabled to construct his inlay from start to finish without trying it in the mouth at any stage, and without delay or inconvenience to the patient.—W. D. Tracy, January, 1907, *Cosmos*.

**Phenol and Camphor in Suppurations.**

The January, 1907, *Cosmos* prints an abstract of a paper by Dr. C. Ehrlich, of Berlin, on Phenol and Camphor as an antiseptic dressing. During the last quarter of the year 1905, the author tested the mixture in a number of cases of whitlow phlegmon, ulcers of the lower limbs, fissure of the anus, furuncles, erysipelas, tuberculous fistulae, and infected wounds of all varieties. By comparing the results obtained with the carbolic acid-camphor mixture with those obtained in similar cases with the antiseptic agents ordinarily employed, Dr. Ehrlich has convinced himself of the fact that applications of carbolic acid-camphor mixtures are more efficacious than dressings with any of the ordinary agents employed for the purpose, and particularly so in cases of acute suppuration. All the whitlow cases, which otherwise require from eighteen to nineteen days to reach the stage of absolute cure, healed satisfactorily under the influence of the mixture under consideration in about six days; and ordinary phlegmons in four days on an average, as against eight days under the usual mode of treatment. Dr. Ehrlich employs a mixture of pure carbolic acid one ounce, alcohol two and a half drams, and camphor two ounces. This mixture differs from that suggested by Dr. Chlumsky in the presence of the alcohol, as the latter's formula is composed exclusively of carbolic acid and camphor. In the application of the carbolic acid-camphor mixture to the treatment of superficial ulcerations and infections, care should be exercised not to employ any form of impermeable dressing material.

**Putrid Canals.** Formaldehyde seems to be the most adaptable remedy. It is a reliable disinfectant and germicide, will not stain and has no unpleasant odor. Used alone it has two objections—it is irritating and very volatile. Both are serious objections, but can be overcome by combining with another drug. If it can be combined with a drug that will render its action slower and more permanent we shall have an ideal agent. The phenols, including carbolic acid, creosote, guaiacol, as well as tricresol, are all objectionable on account of their persistent disagreeable odors, but thymol seems to be just what is wanted. It is like formaldehyde, composed of C. H. O., formula being $C_{20}\ H_{14}\ O_2$ while formaldehyde is $C\ H_2\ O$.

It is difficult to combine thymol with formaldehyde, but it was finally accomplished, and the combination seemed ideal for the purpose. It presented in the form of a crystalline mass, nearly white in color, neutral in reaction, slightly soluble in water, freely soluble in alcohol or the essential oils. The formalin mixed with zinc oxide in the proportion of a 5% compound, and this powder was made into a paste, using glycerine with 5% Thymol in solution as a menstrum. The combination will be an unctious mass, white in color, very slightly soluble in water, which allows it to be used in cases where it was impossible to apply the rubber dam. It can be used as a dressing for disinfecting foul pulp canals and apparently renders them sweet and clean—being sealed in for from one day to a week. It has been tried in cases of chronic dento-alveolar abscess with fistula, filling roots to foramen and in the great majority of cases the fistulae disappear within a reasonable time.

In cases of blind abscess cures were affected apparently by resolution or absorption after the pulp chamber had been lightly filled with the dressing, the time varying from a week to a month. Some obstinate cases of this class were cured after being abandoned as incurable by other methods. Cases where there were bony tumors in the apical region yielded when roots were filled with this dressing. Temporary teeth were treated, and pulp chambers filled, leaving roots empty. In such cases the gums assumed a healthy appearance, and fistulae disappeared. These cases were watched and always the process of shedding and erupting proceeded favorably.

It has been used as a mummifying agent in both the permanent and temporary teeth, removing the pulp from the chamber and, without entering the roots, filling the chamber and tooth at once. It has been used when the root has been found perforated—sealing the perforation with gutta-percha and filling the pulp canal as usual.

Sometimes, like everything else, it will fail, but in the great majority of cases it will disinfect, sterilize, or render the once septic pulp canal aseptic, and thus by removing the cause, effect a permanent cure.—George T. Baker, in October 1906, *Journal.*

**Facial Mal-Development.** I wish in the first place to disclaim any special reputation as a rhinologist or of having any special knowledge upon the subject discussed by Dr Bogue. However, it is of great importance and was first brought to my attention about ten years ago. As we go about the street we recognize that no two men look alike. One has a long nose and another a short one. One a big mouth and another a little one; and we accept these conditions as "natural." Yet in our scientific work we have made very little application of this knowledge. When a child is born with a hare lip or a club foot we do not hesitate to say that it is a developmental defect; but we have entirely failed to recognize the partial failures of Nature. An experience of my own, which, by the way, was a pure accdent, may be of interest in connection with the points made by Dr. Bogue, in showing that the correction of these cases may effect not merely the teeth, but the nasal passages. It happened to me some years ago to have a daughter who, during her second dentition, developed the habit of mouth breathing. With a parent's stupidity I scolded her, telling her to keep her mouth shut, and trusted to Providence that it might pass away. As this did not occur I examined her later and found the septum badly twisted and of course I said to myself: "Some day saws and chisels in this little girl's nose;" but I let it go from day to day until it ran over a year, when a rapidly increasing irregularity of the teeth suddenly forced me to admit that something must be done at once to save her beauty. After consulting one or two dentists and receiving advice that did not satisfy me, I determined to make the change in her teeth myself. The lateral incisors were back of the centrals and set under them for about half their width. I went upon the theory that in order to get room for these teeth the arch must be spread, although I had been told that I must not separate the maxillary bones. I adopted as my method of spreading the arch the jack screw as being the most effective and best under control. One day she came to me saying that there was something in the roof of her mouth and asking what it was. She said it felt like a cut. I examined and found that the bones had been separated. Then I was ready to move the teeth, which I did by means of different applications of the force of the screw. A little later she came to me asking how it happened that she breathed so much more easily. Taking her to my dark room I found that the septum had slipped into its position between the maxillary bones and that it was perceptibly straightened. In the separating of the arches there had been a broadening of the nasal cavities and a mouth breathing child has been changed into one who keeps her mouth closed in breathing. Numbers of similar cases of deflected or distorted nasal septa are of developmental origin and can be corrected by manipulation of the maxillary bones. But one such case does prove that there may be others, and compels us to recognize the possibility of their cure by manipulation instead of mutilation.—C. E. Quimby, in October, 1906, *Journal.*

**The Nobel Prizes.** It is interesting to observe that this year's award of the Nobel prize in medicine has been equally divided between Professor Camillo Golgi, of Italy, and Professor Ramon y Cajal, of Spain. It is of further interest to know that for the first time in its history that portion of the prize which is allotted to the person who has done most for "the formation and propagation of peace congresses" has been awarded to an American, viz.' our own President, Roosevelt, and every one, of course, is *de-lighted*! The important work of Golgi consists in having discovered certain special stains for brain tissue, known since by his name; and Ramon y Cajal's discoveries consisted in employing the great Italian's methods for his fundamental work on embryonic nerve tissue. In the domain of certain branches of medicine—for instance, surgery and hygiene—and in dentistry the United States at present leads the world, and it should not be a surprise if in the near future the prize in medicine of the great Swedish philanthropist's foundation may find its way over the "big pond."

It may be of interest to our readers to know some details of this world-famous institution. We append, therefore, the following short sketch:

"Alfred Nobel bequeathed nearly the whole of his fortune (more than five million dollars) to a fund, the interest on which is annually paid out to those who during the immediate past have conferred the greatest benefit on mankind in certain lines. The interest is to be divided into five equal parts, and to be allotted as follows: One part to the person who shall have made the greatest discovery or invention in the domain of physics; one part to the person who shall have made most important chemical discovery or improvement; one part to the person who shall have made most important discovery in the domain of physiology or medicine; one part to the person who shall have produced in the field of literature the most distinguished work of an idealistic tendency; and one part to the person who shall have most or best promoted the fraternity of nations and the abolition or diminution of standing armies and the formation and propagation of peace congresses. The prizes in physics and chemistry are awarded by the Academy of Science in Stockholm; in physiology and medicine by the Caroline Institute in Stockholm; in literature by the Swedish Academy in Stockholm; and for the work of peace by a committee of five persons nominated by the Norwegian Storthing.

"On an average, each of the five prizes amounts annually to about $40,000. To become a candidate for one of these prizes it is necessary to be proposed in writing by a person competent thereto. The right of proposing a candidate for a prize is held by both Swedish and foreign champions of culture, in accordance with minute instructions issued by the corporations charged with adjusting the prizes, and the proposal must be accompanied by those works and other documents that are cited."

From the foregoing it will be seen that every one who will do some real, hard work in science has an equal chance to participate in the prize; and dentistry, the youngest of the pure sciences, will have as good a showing as any other branch of medicine. *Get busy.—Dental Era.*

**Gold Inlay.** I have found that platinoid, No. 30 to 28 gauge, makes a most excellent cup or form for the retention of the impression material, after preparing the tooth with suitable draft for an impression and the proper setting of an inlay. The platinoid can be easily cut to shape with an ordinary pair of scissors, and bent so as to conform to the contour of the particular requirements. The "Perfection" compound is used, as manufactured by the Detroit Mfg. Co. It is first rolled into pencil or stick form, which permits of cutting after slight warming. The platinoid having been cut and bent, it should be heated in the alcohol flame, so that the required amount of the compound can be made to adhere perfectly to its surface, so that when withdrawing the impression the two will come away together.

In cases that have been protected by the rubber dam—the cavity of course having been prepared with suitable draft and coated with vaselin—the cup and compound may be removed without the slightest danger of any distortion.

Having secured an accurate impression in this way, it is invested in plaster, the surfaces of which are exposed, that amalgam may be packed and burnished into the mold, resulting in a practically perfect reproduction of the cavity. The amalgam should be mixed thin, and the mercury worked out as you are filling in the mold, and allowed to stand over night. This amalgam die is taken out of the plaster and embedded in a composition called "dental lac" contained in one of the cups which belong to the Brewster so-called water-press.

Now adjust a piece of pure gold of No. 34 gauge over the die, press it down into the cavity with a piece of spunk, and burnish. Take it out, trim and anneal it, and replace on the amalgam die. Put a piece of spunk in the center, place in the press and swage, and trim to about 1 mm. of the cavity margin; then anneal and swage again.

Fill the matrix with any quick-setting cement and place it in the cavity and instruct the patient to bite while the cement is still soft. Take it out and trim to contour, and around the edge trim below the enamel margin to about the thickness of the piece of gold that you are to swage for the top cap. Replace it on the amalgam die and swage the top piece of No. 36 gauge pure gold; trim away to the line where you are to unite the two pieces, anneal, and re-swage.

I separate the two pieces, and usually find upon tapping the matrix that the cement will drop out very readily; if not catch the matrix with the foil-carriers, and tap them once or twice. The cement will drop out whole, so that you may have it at any time to straighten out the cap if distorted in any manner.

I cut out of the matrix one-half or more of its center, keeping away from its margin in accordance with the shape of the cavity. In cutting this hole the matrix becomes somewhat distorted; I anneal it, replace it in the amalgam die, and burnish well to place; take out again and anneal, place the cement core in position, and re-swage; remove the cement core, and unite the two pieces over the Bunsen flame—at one point only—with 22-k. solder and a little powdered borax. Try it in the amalgam die, and see that the two pieces have a close contact all around. Then finish with 22k. solder and fill in with any low-karat solder, leaving enough space inside to act as an undercut in retaining the filling.

Set it in the tooth and allow the cement to harden. All that now remains to be done is to run a disk over the edges, and polish with a brush wheel.

I have found this method applicable to the deciduous as well as to the permanent teeth, and particularly advantageous in that it eliminates the long and tedious operations necessary for the introduction of gold foil. It also eliminates or relieves a child of the fatigue and discomfort of unnecessary long time with the rubber dam in position.—W. B. Dills, January, 1907, *Cosmos*.

**Light for the Dentist.** In any event the *direct* rays of the sun should not be allowed to shine into the mouth of the patient, not only because the teeth give reflections that dazzle the operator, but because in using the mirror an entirely too strong illumination is the result.

While we are on the subject of mirrors I suppose they cannot be dispersed with, and yet I regard their use as one of the principal sources of eye-strain in dental work. While it is true that during the working period the light is most of the time simply reflected upon the teeth and oral walls it is, now and then, flashed into the eyes of the operator to the distinct detriment of these organs. That is to say, the dentist is straining his eye to see microscopal objects and at the same time throwing strong light upon his tense choroid just as—to compare small things with great—one runs an "obstruction" race.

The *wall tints of the operating room* have an ocular value, and should vary according to the kind and amount of the general illumination. If a north (or west) light be generally used dull cream, light buff, or light green paper, paint, calcimine or burlap may be used with benefit. If the light be from the east or south, still darker tints are unobjectionable. In other words, while always using soft, *unglazed* colors these may be made to vary with the intensity of the light admitted to the operating room—the brighter the illumination the darker the wall tints. Let me repeat, glazed paper, or other objects that strongly reflect light into the eyes of the operator should be banished from the dentists's operating room.

You may easily imagine, then, my opinion of the method of *illuminating the oral cavity employed by Dr. Bing, of Paris.* As he operates in a dark room, the only light admitted being that conveyed by means of a movable tube to the face of the patient. In my judgment this is an ideal arrangement so far as the eyesight of the dentist is concerned. There may be extrinsic objections to it, but it affords, in great measure, the best form of illuminating the mouth with the least expenditure of energy by the ocular apparatus.

The *use of the monocle* or loupe in dental work is, to my mind, objectionable. It usually argues the possession by the dentist of defective eyes, defective eye muscles, or incorrect glasses. There is, in other words, generally something wrong with his binocular vision when the operator is driven to the use of a magnifying glass before one eye only. Such a device deprives him of stereoscopic vision and is, in several ways, a source of strain to both eyes. Better consult an oculist and see if the total visual capacity cannot be more satisfactorily increased by improving his binocular vision.

The *light from Luxfer prisms*, or similar devices, is not satisfactory in dental work. These prism "batteries" are of great value in lighting dark basements or the interior rooms of city buildings, but they have no place in the dental operating chambers that should always be outside or corner rooms with a good sky exposure.

The same judgment must be passed upon *cross lights*. The illumination of the dental chair should be, preferably, from one window placed above the level of the patient's face. Equally effective, of course, is it to cover the lower part of a deep window with an opaque shade allowing only the light from the upper portion to shine upon the field of operation. In other words, the best effects are obtained from a sufficient light falling from a comparatively small aperature upon the mouth of the patient, the rest of the room, as well as the dentist's face being in comparative darkness.

The question whether the dentist's glasses should be worn constantly or only when he is doing near work, will entirely depend upon a number of considerations. If he is decidedly astigmatic or far-sighted, or has weak eye muscles, or is not in the best of health, or suffers from eye symptoms due to strain when going about, he ought to use these aids to comfortable vision all the time. If, however, there is no strain upon the ocular apparatus or nervous system except during periods of close work, then near glasses are all that he needs. —Dr. Casey Wood, in December, 1906, *Review*.

